# Supplementary material for: Neutralization of SARS-CoV-2 variants by convalescent and BNT162b2 vaccinated serum
Source: Nat Commun. 2021 Aug 26;12:5135. doi: 10.1038/s41467-021-25479-6 (PMC8390486; doi:10.1038/s41467-021-25479-6)
Supplement: Supplementary file 1 — Supplementary Information [file 41467_2021_25479_MOESM1_ESM.pdf]

Supplementary Information

1) **Supplementary Table 1 -** List of mutations in B.1.1.7 and B.1.351 SARS-CoV-2 clinical isolates
2) **Supplementary Table 2 -** List of primers used in this study
3) **Supplementary Figure 1** – Vaccinee serum FRNT50 curves
4) **Supplementary Figure 2 –** Convalescent serum FRNT50 curves
5) **Supplementary Figure 3 –** Vaccine response by sex
6) **Supplementary Figure 4 –** Correlates of selected demographic and clinical factors with neutralization in the COVID-19 convalescent cohort
7) **Supplementary Figure 5 –** Variant focus forming assay phenotypes

Supplementary Table 1. List of mutations in B.1.1.7 and B.1.351 SARS-CoV-2 clinical isolates*

Lineage	GISAID Clade	GISAID ID	Spike mutations	Non-Spike mutations
B.1.1.7	GR	EPI_ISL_683466	H69del, V70del, Y145del, #N501Y, A570D, D614G, P681H, T716I, S982A, D1118H	N D3L, N G204R, N R203K, N S235F, NS8 Q27stop, NS8 R52I, NS8 Y73C, NSP3 A890D, NSP3 A1305V, NSP3 I1412T, NSP3 T183I, NSP6 F108del, NSP6 G107del, NSP6 S106del, NSP12 P323L, NSP13 K460R, NSP14 E347G
B.1.351	GH	EPI_ISL_678570	D80A, D215G, L242del, A243del, L244del, #K417N, #E484K, #N501Y, D614G, Q677H**, A701V	E P71L, N T205I, NS3 Q57H, NS3 S171L, NSP2 T85I, NSP3 K837N, NSP5 K90R, NSP6 F108del, NSP6 G107del, NSP6 S106del, NSP12 P323L

Mutation within the RBD

* Obtained from BEI Resources

** Additional mutation not found in clinical isolate

Supplementary Table 2. List of primers used in this study

Oligo	Sequnce
M13 reverse (pCAGGS sequencing primer)	CAGGAAACAGCTATGAC
Tn5-i5-Adapter	TCGTCGGCAGCGTCTCCACGC [i5-Tn5-Index] GATCGAGGACGGCAGATGTGTATAAGAGACAG
Tn5-i7-Adapter	GTCTCGTGGGCTCGGCTGTCCCTGTCC [i7-Tn5-Index] CCGTCTCCGCCTCAGATGTGTATAAGAGACAG
Tn5-ME	5Phos/CTGTCTCTTATACACATCT
PCR-i5-Primer	AATGATACGGCGACCACCGAGATCTACAC [i5-PCR-Index] TCGTCGGCAGCGTC
PCR-i7-Primer	CAAGCAGAAGACGGCATACGAGAT [i7-PCR-Index] GTCTCGTGGGCTCGG
Read 1 Sequencing Primer	GATCGAGGACGGCAGATGTGTATAAGAGACAG
Read 2 Sequencing Primer	CCGTCTCCGCCTCAGATGTGTATAAGAGACAG
Index 1 Sequencing Primer	CTGTCTCTTATACACATCTGAGGCGGAGACGG
Index 2 Sequencing Primer	CTGTCTCTTATACACATCTGCCGTCCTCGATC

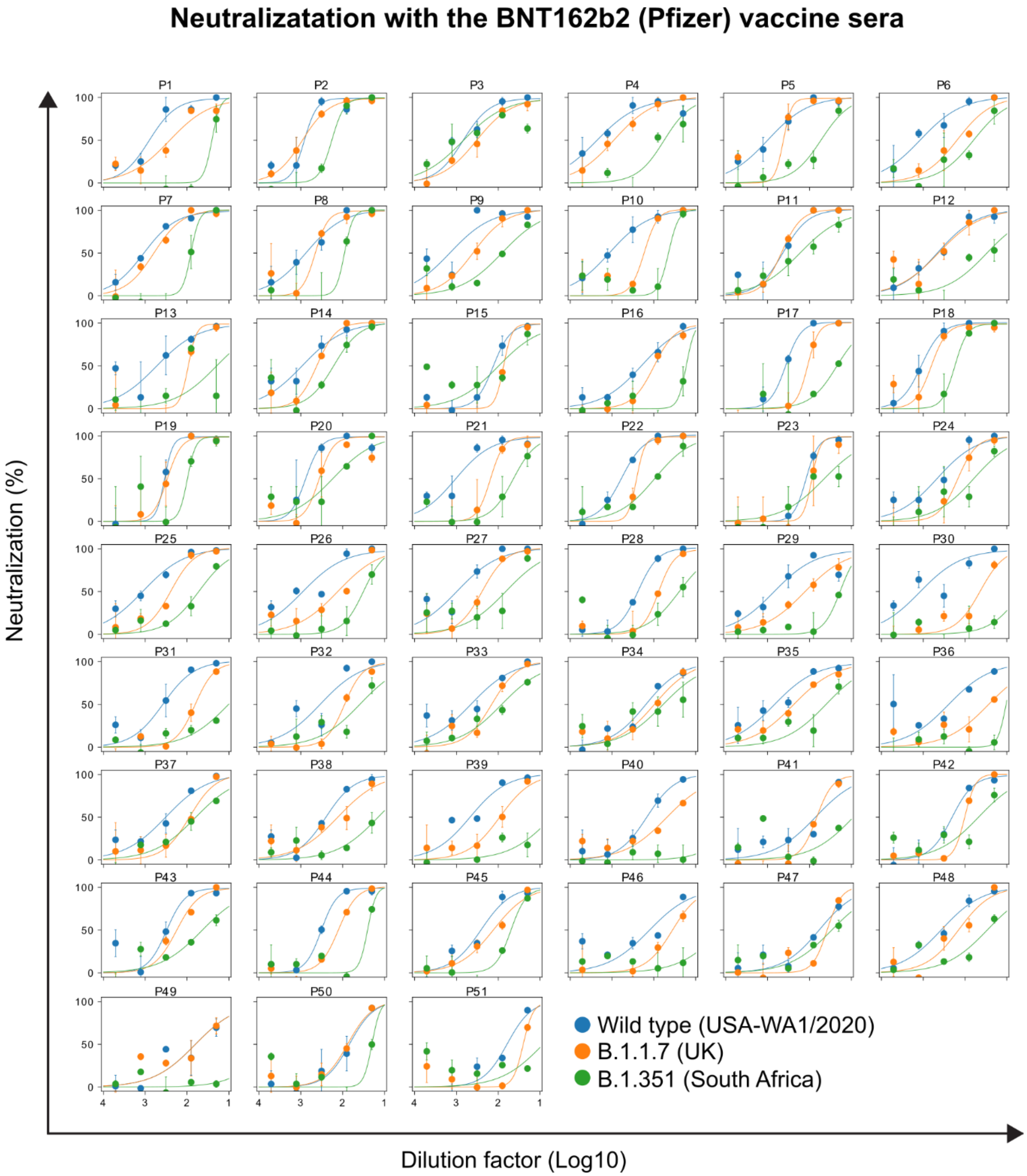

Supplementary Figure 1. Vaccinee serum FRNT50 curves. Neutralization curves of serum (n = 51) against the different strains of SARS-CoV-2 are shown. Serum was collected two weeks after the second dose of the BNT162b2 vaccine. Error bars represent SEM of biological replicates.

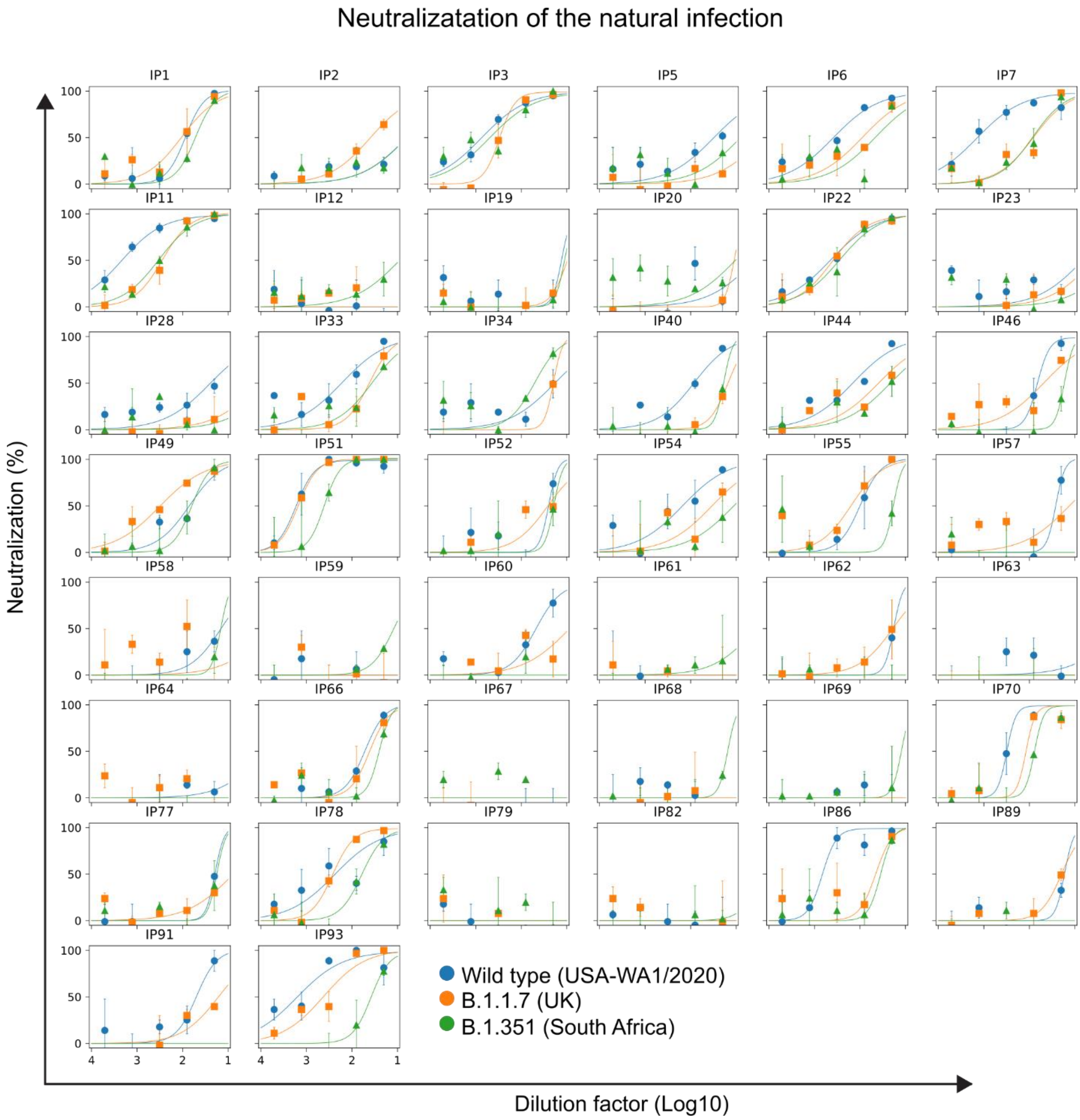

Supplementary Figure 2. Convalescent serum FRNT50 curves. Neutralization plots of convalescent sera (n = 44) against the different strains of SARS-CoV-2 are shown. Error bars represent SEM of biological replicates.

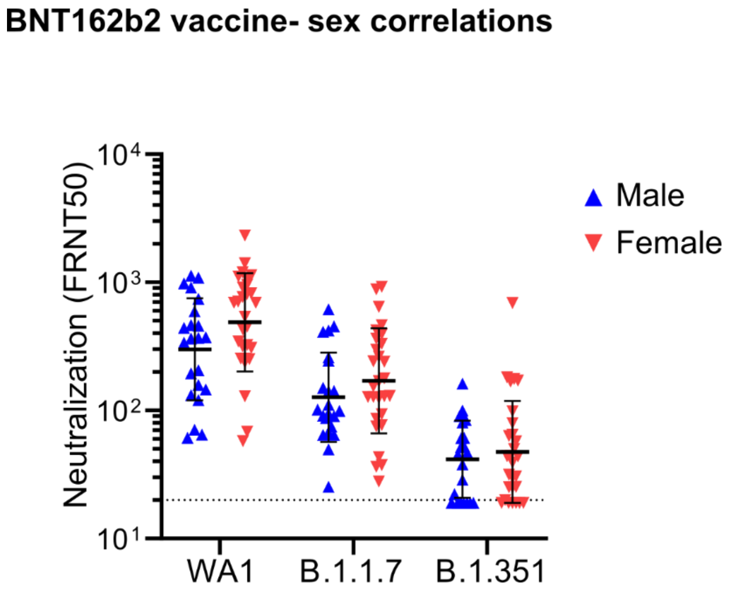

Supplementary Figure 3. Vaccine response by sex. Comparison of vaccine sera neutralization titers (FRNT50) of the different SARS-CoV-2 strains showing no correlation with sex. The male group contains n=22, and the female group contains n=28 independent biological samples. Data are presented as the geometric mean +/- SD with individual values shown. Statistical comparison was performed using a two-way ANOVA with the Šidák multiple comparison correction. There is no significance correlation between vaccine serum neutralization titers with sex.

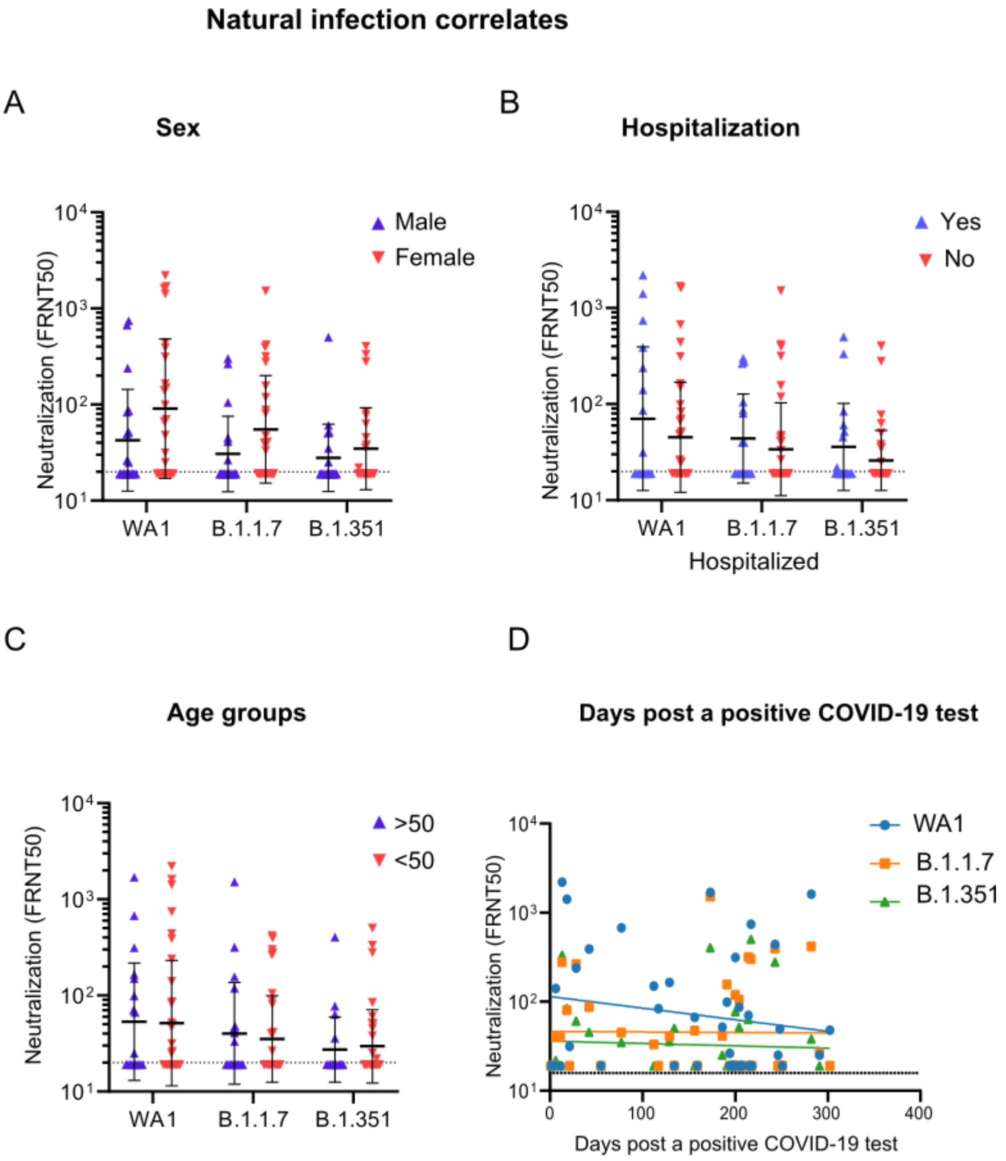

Supplementary Figure 4. Correlates of selected demographic and clinical factors with neutralization in the COVID-19 convalescent cohort. A-C) Correlation of convalescent neutralization titers with sex (male n=19, female n=25 biologically independent samples) (A), hospitalization versus ambulatory care (yes n=17, no n=37 biologically independent samples) (B), and age of COVID-10 patients (>50 n=19, <50 n=35 biologically independent samples) (C). Data are presented at the geometric mean +/- SD with individual values shown. D) comparison of FRNT50 and the number of days between the date of

confirmatory COVID-19 PCR test and the date of serum sample collection. Statistical comparisons were performed using a two-way ANOVA with the Šidák multiple comparison correction. There is no significant correlation between convalescent neutralization titers and sex, hospitalization, age or days after a positive COVID-19 test.

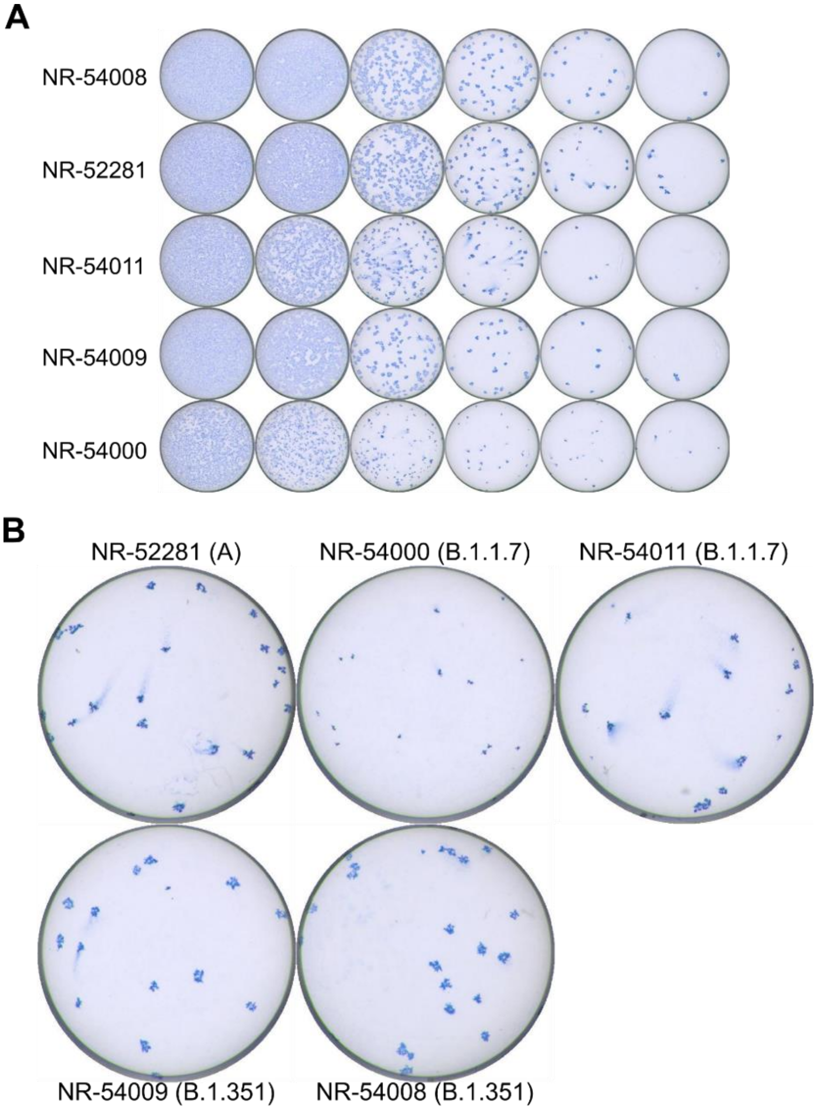

C

Lineage	BEI number	Average focus size (pixels)
A	NR-52281	193.7
B.1.1.7	NR-54000	51.9
B.1.1.7	NR-54011	212.8
B.1.351	NR-54009	303.8
B.1.351	NR-54008	309.4

Supplementary Figure 5. Variant focus forming assay phenotypes. Focus assay well images showing an example of the utilized titration curves for the clinical isolates tested during for assay development (A). Increased resolution of wells with individual foci (B). Average focus size for each isolate (C). Individual focus sizes were measured manually using ImageJ using the images

indicated in (B). The average size indicates the mean number of pixels across all foci in each image, excluding those contacting the edge of the well.
