# Supplementary material for: Neutralization of SARS-CoV-2 variants by convalescent and BNT162b2 vaccinated serum
Source: Nat Commun. 2021 Aug 26;12:5135. doi: 10.1038/s41467-021-25479-6 (PMC8390486; doi:10.1038/s41467-021-25479-6)
Supplement: Supplementary file 3 — Reporting Summary [file 41467_2021_25479_MOESM3_ESM.pdf]

nature portfolio

Corresponding author(s): Fikadu G. Tafesse, William B. Messer, Marcel E. Curlin

Last updated by author(s): Aug 4, 2021

Reporting Summary

Nature Portfolio wishes to improve the reproducibility of the work that we publish. This form provides structure for consistency and transparency in reporting. For further information on Nature Portfolio policies, see our Editorial Policies and the Editorial Policy Checklist.

Statistics

For all statistical analyses, confirm that the following items are present in the figure legend, table legend, main text, or Methods section.

n/a	Confirmed	
☐	☒	The exact sample size (*n*) for each experimental group/condition, given as a discrete number and unit of measurement
☐	☒	A statement on whether measurements were taken from distinct samples or whether the same sample was measured repeatedly
☐	☒	The statistical test(s) used AND whether they are one- or two-sided<br>*Only common tests should be described solely by name; describe more complex techniques in the Methods section.*
☒	☐	A description of all covariates tested
☐	☒	A description of any assumptions or corrections, such as tests of normality and adjustment for multiple comparisons
☐	☒	A full description of the statistical parameters including central tendency (e.g. means) or other basic estimates (e.g. regression coefficient) AND variation (e.g. standard deviation) or associated estimates of uncertainty (e.g. confidence intervals)
☐	☒	For null hypothesis testing, the test statistic (e.g. F, t, r) with confidence intervals, effect sizes, degrees of freedom and P value noted<br>*Give P values as exact values whenever suitable.*
☒	☐	For Bayesian analysis, information on the choice of priors and Markov chain Monte Carlo settings
☒	☐	For hierarchical and complex designs, identification of the appropriate level for tests and full reporting of outcomes
☒	☐	Estimates of effect sizes (e.g. Cohen's d, Pearson's r), indicating how they were calculated

Our web collection on statistics for biologists contains articles on many of the points above.

Software and code

Policy information about availability of computer code

Data collection	No software was used for data collection
Data analysis	atient sample randomization was performed using R version 4.0.3 in Rstudio version 1.2.5001; Focus counts were obtained using CTL ImmunoSpot Professional DC version 7.0.26.0. EC50 and FRNT50 values were calculated using python version 3.7.6 with numpy version 1.18.1, scipy version 1.4.1, pandas version 1.0.1, and matplotlib version 3.1.3 libraries. Statistical comparisions were performed using Graphpad Prism version 9.0.2.

For manuscripts utilizing custom algorithms or software that are central to the research but not yet described in published literature, software must be made available to editors and reviewers. We strongly encourage code deposition in a community repository (e.g. GitHub). See the Nature Portfolio guidelines for submitting code & software for further information.

Data

Policy information about availability of data

All manuscripts must include a data availability statement. This statement should provide the following information, where applicable:

- Accession codes, unique identifiers, or web links for publicly available datasets
- A description of any restrictions on data availability
- For clinical datasets or third party data, please ensure that the statement adheres to our policy

The data associated with this study are provided in the supplementary materials or in public repositories. The SARS-CoV-2 clinical isolate deep sequencing data generated in this study have been deposited in Genbank under accession codes found here: WA1/2020 consensus: MZ344995; B.1.1.7 consensus: MZ344998; B.1.351 consensus: MZ344999, raw sequence reads: PRJNA742050. The raw EC50 and FRNT50 curves are provided in the Supplementary Information (Figures S1, S2). Raw focus counts and calculated FRNT50 and EC50 values are available on Zenodo at DOI: 10.5281/zenodo.5157016. The FRNT50 calculation code used in this

study is available on Github at DOI: 10.5281/zenodo.5158655.

Field-specific reporting

Please select the one below that is the best fit for your research. If you are not sure, read the appropriate sections before making your selection.

- [x] Life sciences
- [ ] Behavioural & social sciences
- [ ] Ecological, evolutionary & environmental sciences

For a reference copy of the document with all sections, see nature.com/documents/nr-reporting-summary-flat.pdf

Life sciences study design

All studies must disclose on these points even when the disclosure is negative.

Sample size	Sample size was determined based on sample availability and biosafety level 3 laboratory throughput; they were designed to exceed existing studies utilizing similar methods (DOI: 10.1056/NEJMc2102017).
Data exclusions	FRNT50 measurements of patient sera on individual viruses were excluded based on pre-established criteria. FRNT50 values were calculated for each replicate separately, and when a sample had replicates with more than 4-fold difference, it was excluded.
Replication	One sample was excluded based on the above criteria. All other attempts at replication were successful.
Randomization	Experimental groups were assigned based on vaccination status (for the vaccination cohort) or previous infection status (for the convalescent cohort). Samples were selected from a larger cohort to maintain equal sex and age distributions between experimental and control groups. Further details of inclusion criteria can be found in the methods section.
Blinding	Investigators were blinded from sample demographic data during laboratory experiments and data analysis until completion of FRNT50 and EC50 calculations.

Reporting for specific materials, systems and methods

We require information from authors about some types of materials, experimental systems and methods used in many studies. Here, indicate whether each material, system or method listed is relevant to your study. If you are not sure if a list item applies to your research, read the appropriate section before selecting a response.

Materials & experimental systems

n/a	Involved in the study	
[ ]	[x]	Antibodies
[ ]	[x]	Eukaryotic cell lines
[x]	[ ]	Palaeontology and archaeology
[x]	[ ]	Animals and other organisms
[ ]	[x]	Human research participants
[x]	[ ]	Clinical data
[x]	[ ]	Dual use research of concern

Methods

n/a	Involved in the study	
[x]	[ ]	ChIP-seq
[x]	[ ]	Flow cytometry
[x]	[ ]	MRI-based neuroimaging

Antibodies

Antibodies used	anti-human GOXHU IgG/A/M HRP [Invitrogen, Cat#A18847, lot:69-154-042020]; anti-Llama HRP [Novus, #NB7242, Lot: P20]; anti-SARS-CoV-2 spike RBD alpaca serum was generated by three repeated boosts of an alpaca with recombinant SARS-CoV-2 spike protein.
Validation	anti-human GOXHU IgG/A/M HRP [Invitrogen, Cat#A18847, lot:69-154-042020] was validated by the manufacturer for use in ELISA, and we evaluated its performance with positive and negative control samples in our ELISA. Use of this antibody for this assay has been previously published (DOI:10.1001/jama.2021.11656).<br><br>anti-Llama HRP [Novus, #NB7242, Lot: P20] was validated by the manufacturer for use in immunocytochemistry, and we determined the optimal concentration for our focus forming assay (supplemental figure S5). Use of this antibody for this assay has been previously published (DOI:10.1001/jama.2021.11656).<br><br>The anti-SARS-CoV-2 spike RBD alpaca serum was validated by immunofluorescence, western blot, and neutralization experiments in a previously published report (DOI:10.1016/j.celrep.2021.108737). Use of this polyclonal serum for this assay has been previously published (DOI:10.1001/jama.2021.11656).

Eukaryotic cell lines

Policy information about cell lines

Cell line source(s)	Vero E6 cells were obtained from ATCC (CRL-1586). HEK 293F cells were obtained from ThermoFisher (Cat no. R79007)
Authentication	Authenticated Vero E6 and 293F cell lines were obtained from the manufacturers. We did not perform additional authentication, and all cells were used at low passage.
Mycoplasma contamination	All cells tested negative for mycoplasma contamination using the ATCC Universal Mycoplasma Detection Kit (Cat no. 30-1012K)
Commonly misidentified lines (See ICLAC register)	No commonly misidentified cell lines were used in this study

Human research participants

Policy information about studies involving human research participants

Population characteristics	The convalescent cohort (n=50) had a median age of 56 (range 1-88), was 58% female, 92% had symptomatic infection, 32% were hospitalized, 9.25% were admitted to the ICU, and had a median time between infection and sample donation of 188.5 (range 1-302). The vaccinated cohort (n=51) had a median age of 50 (range 21-82), was 54.9% female, had median time between doses of 21 days (range 20-22), and a median time between second dose and sample donation of 14 days (range 14-15).
Recruitment	Convalescent cohort subjects were enrolled at the time of COVID-19 PCR testing. We do not believe any self selection bias affected the results. Vaccinee cohort subjects were enrolled during their first vaccine visit. All vaccinee cohort members were employees of OHSU and are either healthcare workers, administrators, or researchers. We do not believe that this self-selection bias impacted the final results of the study.
Ethics oversight	This study was performed with approval from the Oregon Health & Science University Institutional Review Board and complies with all state and federal regulations. Written informed consent was obtained from all participants. The IRBs (#00022511 and #21230) are on file with the corresponding authors and at OHSU.

Note that full information on the approval of the study protocol must also be provided in the manuscript.
